# Beyond the Lungs: A Case Report of Disseminated Tuberculosis With Multisystem Involvement

**DOI:** 10.7759/cureus.78484

**Published:** 2025-02-04

**Authors:** Nuno Carvalho, André Pereira, Margarida Castro, Olinda Miranda, Margarida Rocha, Magda Fernandes, Carlos Fernandes, Jorge Cotter

**Affiliations:** 1 School of Medicine, University of Minho, Braga, PRT; 2 Internal Medicine, Hospital da Senhora da Oliveira, Guimarães, PRT; 3 Cardiology, Hospital da Senhora da Oliveira, Guimarães, PRT

**Keywords:** acid-fast bacillus, disseminated tuberculosis, extrapulmonary tuberculosis, miliary tuberculosis, mycobacterium tuberculosis, pulmonary silicosis, tuberculosis

## Abstract

Tuberculosis (TB) is an infectious disease caused by acid-fast bacillus pertaining to the *Mycobacterium tuberculosis *complex. Pulmonary TB is the most common presentation, resulting either from primary infection or reactivation of latent disease. In rare cases, wide dissemination of *M. tuberculosis* can occur, usually by hematogenous or lymphatic route, leading to multiorgan involvement and potentially life-threatening conditions known as disseminated TB. We present the case of a 55-year-old man who presented to the emergency department (ED) with complaints of inflammatory polyarthralgia and myalgia, gradually worsening in the last four months. Other symptoms included fatigue, cough with purulent sputum, and weight loss within the last month. The patient’s past medical history included pulmonary silicosis and tobacco use. On physical examination, he had an emaciated appearance, fever (38.4 ºC), normal thoracic examination, and no evidence of arthritis. Blood tests displayed anemia, leucopenia, mild hepatic cytolysis, and elevated acute phase reactants. Urine sediment revealed mild hematuria with red blood cell casts. A thoraco-abdominal-pelvic computerized tomography scan revealed diffuse ground-glass peribronchovascular densification, left pleural effusion, homogenous hepatosplenomegaly, and multiple mediastinal, retroperitoneal, periportal, iliac, and inguinal lymphadenopathy. After admission, polymerase chain reaction (PCR) of *M. tuberculosis* DNA was positive in sputum and urine. Disseminated TB, with pulmonary and renal involvement, was diagnosed, and antituberculous therapy was initiated with isoniazid, rifampicin, pyrazinamide, and ethambutol. Additionally, 24-hour urine was collected, and proteinuria of 1,566 mg/24 hour was evident. An ultrasound-guided percutaneous kidney biopsy was performed, revealing mesangioproliferative glomerulonephritis due to immune complexes deposition. Polyarthralgia persisted with new-onset arthritis, so arthrocentesis was performed. Both mycobacteriology and PCR detection of *M. tuberculosis* DNA were negative. While in the ward, sudden onset dyspnea with lower limb edema developed, and jugular vein distention with hypotension was detected. Point-of-care cardiac ultrasound revealed a large volume of pericardiac effusion without cardiac tamponade. Ultrasound-guided pericardiocentesis was performed. Pericardial fluid’s mycobacteriology and PCR detection of *M. tuberculosis* DNA were negative. Six weeks after admission, *M. tuberculosis* was identified in Lowenstein-Jensen cultures of sputum. The patient was discharged after 145 days of hospital stay, with an indication to maintain antituberculous treatment for a minimum of 12 months, with prolonged treatment decisions dependent on clinical evolution. Twelve months after discharge, the patient was asymptomatic, with analytical and imagiological improvement; therefore, antituberculous therapy was discontinued. Disseminated or miliary TB is a rare condition that poses a diagnostic challenge for every clinician, as clinical presentation is non-specific. Multiorgan involvement may impair diagnostic workup if TB is not initially suspected. Clinicians should be aware of heterogeneous disease progression, as initial detection of organ involvement does not exclude possible further disseminated disease. Diagnosis should be swift to allow early antituberculous therapy initiation and prevent potentially life-threatening situations.

## Introduction

Tuberculosis (TB) is an infectious disease caused by acid-fast bacillus pertaining to the *Mycobacterium tuberculosis *complex. It remains one of the most significant global health challenges worldwide. The World Health Organization (WHO) estimates that 8.2 million new TB cases will be diagnosed and notified in 2023, the highest number since data records began in the mid-1990s [1]. Epidemiology varies substantially around the world, with incidence rates higher than 300 cases per 100.000 inhabitants being registered in developing countries around sub-Saharan Africa and Asian regions. Meanwhile, lower incidences (less than 25 cases per 100,000 inhabitants) occur in North America, Western Europe, Japan, and Australia [2].

TB terminology is inconsistent in the literature. Newer designations divide patients between TB infection (when there is evidence of specific cell-mediated immunologic response following exposure to *M. tuberculosis*) and TB disease (when signs and symptoms of illness due to *M. tuberculosis* are present) [3].

Pulmonary TB is the most common presentation of TB, accounting for 62% of bacteriologically confirmed diagnoses in 2023, either from primary infection or reactivation of latent disease [1]. In rare cases, widespread *M. tuberculosis* can occur, usually by hematogenous or lymphatic route, leading to multiorgan involvement and potentially life-threatening conditions known as disseminated TB [4,5].

## Case presentation

We present the case of a 55-year-old man who presented to the emergency department (ED) with complaints of polyarthralgia and myalgia. Symptoms started four months before, with inflammatory characteristics and progressive increase in intensity, and were associated with fatigue. Within the last month, a cough with purulent sputum and weight loss (5 kg - approximately 4% of his total body weight) developed. There was no fever, headache, night sweats, dyspnea, hemoptysis, or known insect bites. In addition, he did not have recent contact with ill persons or exposure to dust particles. The patient never traveled or lived in a TB-endemic area. 

The patient’s past medical history included tobacco use (approximately 20 cigarettes per day) and pulmonary silicosis, previously diagnosed in the context of his employment as a miner.

On physical examination, the patient had emaciated appearance, fever (38.4 ºC), normal thoracic examination, and no evidence of arthritis. Other vital signs were within normal range, including peripheral oxygen saturation. Relevant blood tests results are displayed in Table 1, and blood-gas analysis with a fraction of inspired oxygen (FiO_2_) of 21% is displayed in Table 2. Urine dipstick test with sediment analysis is shown in Table 3.

**Table 1:** Blood test results The results display normocytic/normochromic anemia, leucopenia with lymphopenia, hepatic cytolysis, and elevated C-reactive protein. INR: International normalized ratio

Parameters	Results	Reference range
Hemoglobin (g/dL)	11.8	13.0-16.0
Hematocrit (%)	34.9	41-53
Mean corpuscular volume (fL)	85.3	83-103
Mean corpuscular hemoglobin (pg)	28.9	28-34
Leukocytes (x10^3^ /uL)	3.0	4.8-10.8
Neutrophils (x10^3^ /uL)	2.6	1.8-7.7
Eosinophils (x10^3^ /uL)	0.1	0.0-0.49
Basophils (x10^3^ /uL)	0.0	0.0-0.1
Lymphocytes (x10^3^ /uL)	0.4	1.0-4.8
Monocytes (x10^3^ /uL)	0.3	0.1-0.8
Platelet count (x10^3^ /uL)	181	150-350
Urea (mg/dL)	32	15-39
Creatinine (mg/dL)	0.57	0.57-1.11
Aspartate aminotransferase (UI/L)	68	12-40
Alanine aminotransferase (UI/L)	50	7-40
Gamma glutamyl transferase (UI/L)	214	0-73
Lactate dehydrogenase (UI/L)	289	46-116
C-reactive protein (mg/dL)	116	<5
INR	1.0	0.9-1.1

**Table 2:** Blood-gas analysis with FiO2 (21%) Blood-gas analysis shows compensated metabolic acidosis, without hypoxemia.

Parameters	Results	Reference range
pH	7.45	7.35-7.45
pCO_2_ (mmHg)	31	35-45
pO_2_ (mmHg)	83	80-100
HCO_3_- (mmol/L)	21.3	22-31
Lactate (mmol/L)	1.4	0.5-2.2

**Table 3:** Urine dipstick test and sediment analysis A dipstick test is positive for hemoglobin and proteins. Sediment analysis shows mild hematuria with red-blood cell casts.

Parameters	Results	Reference range
pH	5.5	-
Leucocytes	Negative	-
Nitrites	Negative	-
Proteins	++	-
Glucose	Negative	-
Ketone bodies	Negative	-
Urobilinogen	Negative	-
Hemoglobin	Positive	-
Urinary sediment analysis		
Erythrocytes (/uL)	28	<20
Leucocytes (/uL)	8	<28
Epithelial cells (/uL)	5	<28
Red-blood cell casts (/uL)	11	<2

**Figure 1:**
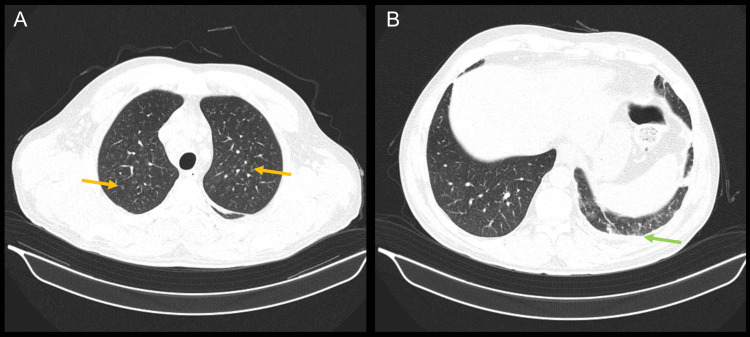
Thoraco-abdominal-pelvic CT scan: lung window (A) Diffuse ground-glass peribronchovascular densifications (yellow arrows). (B) Left pleural effusion (green arrow).

**Figure 2:**
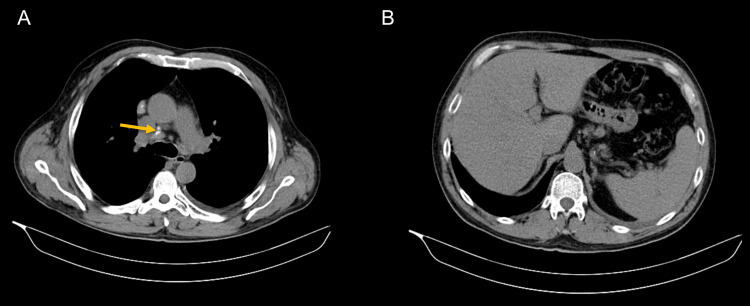
Thoraco-abdominal-pelvic CT scan: soft-tissue window (A) Partially calcified mediastinal lymphadenopathy (yellow arrow). (B) Diffuse hepatosplenomegaly.

Thoraco-abdominal-pelvic computerized tomography scan (CT) revealed diffuse ground-glass peribronchovascular densifications (Figure 1A), left pleural effusion with maximum thickness of one centimeter (Figure 1B), homogeneous hepatosplenomegaly (Figure 2B), as well as multiple mediastinal, retroperitoneal, periportal, iliac, and inguinal lymphadenopathy, the largest with 2 cm in diameter (Figure 2A).

The patient was admitted to the internal medicine ward for an etiologic study. Samples were collected for blood, urine, and sputum cultures. Polymerase chain reaction (PCR) detection of *M. tuberculosis* DNA was performed in urine and sputum. Anti-HIV, HBsAg, anti-HCV, *Mycoplasma pneumoniae*, *Legionella pneumoniae,* and *Chlamydia pneumoniae* serologies were negative. Interferon-gamma release assay (IGRA) was inconclusive, even though retesting was performed. Additional serologic studies, comprising antinuclear antibodies, rheumatoid factor, anti-double-stranded DNA, myositis, and vasculitis panels were negative, excluding inflammatory/autoimmune etiologies.

Flexible bronchoscopy showed no visible macroscopic abnormalities. Bronchoalveolar aspirate (BAA) cytology displayed numerous alveolar macrophages with inflammatory cells, and microbiology/mycobacteriology cultures, as well as DNA detection of *M. tuberculosis*, were negative.

As cough with purulent sputum and fever persisted, empiric antibiotic therapy with amoxicillin/clavulanate was initiated for possible upper respiratory tract infection. After antibiotic course, fever subsided but complaints of polyarthralgia and myalgia persisted.

On the thirteenth day of hospitalization, DNA detection of *M. tuberculosis* was positive in both sputum and urine samples, leading to the diagnosis of disseminated TB with pulmonary and renal involvement. Quadruple antituberculous therapy was initiated with isoniazid, rifampicin, pyrazinamide, and ethambutol with pyridoxine supplementation.

Given microscopic hematuria with red blood cell casts, 24-hour urine was collected, and proteinuria of 1,566 mg/24 hour was evident. An ultrasound-guided percutaneous kidney biopsy was performed with sample collection for immunofluorescence, optic, and electronic microscopy. Subsequent results revealed mesangioproliferative glomerulonephritis due to immune complexes deposition, further supporting renal involvement from disseminated tuberculosis. 

Despite antituberculous therapy initiation, at the third week of inpatient stay, polyarthralgia persisted with new-onset arthritis and effusion. Arthrocentesis from the right knee was performed and an elevated leucocyte count (four times higher than the reference range), with polymorphonuclear cells predominance, was evident, as well as elevated adenosine deaminase value (62.4 U/L for a reference range of 4.8-23.1). No crystals were seen in the synovial fluid. Microbiology, mycobacteriology, and PCR detection of *M. tuberculosis* DNA were negative. Corticosteroid therapy with prednisolone 1 mg/kg was initiated, one month after antituberculous therapy, with subsequent clinical improvement.

**Figure 3:**
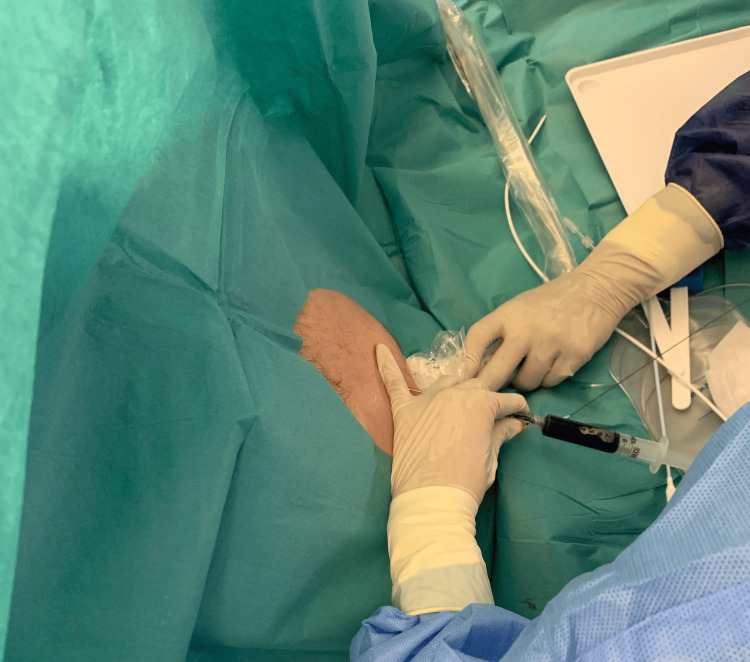
Ultrasound-guided pericardiocentesis Ultrasound-guided pericardiocentesis with collection of serous-hematic pericardial fluid.

During the fifth week of hospitalization, sudden onset dyspnea, hypotension, jugular vein distention, and lower limb edema were detected. Point-of-care cardiac ultrasound evaluation revealed a large volume of pericardiac effusion in the subcostal view (Video 1). There were no clinical or echographic signs of cardiac tamponade. Ultrasound-guided pericardiocentesis was performed (Figure 3), and pericardial fluid samples were collected. Total protein and adenosine deaminase (ADA) levels were within normal range. Direct and cultural mycobacteriology analysis, as well as DNA detection of *M. tuberculosis,* were negative. There were no complications or relapses following pericardiocentesis.

**Video 1:** Subcostal view of pericardial effusion in the point-of-care ultrasound cardiac evaluation In this clip, a large volume circumferential pericardial effusion is visible in a subcostal view.

Six weeks after admission, definitive *M. tuberculosis* growth in Lowenstein-Jensen sputum cultures was identified. Drug-susceptibility testing revealed sensitivity to all antibiotics in course. The patient was discharged after 145 days of hospital stay. Antituberculous treatment for 12 months was planned, with longer treatment dependent on clinical evolution.

At follow-up revaluation, 12 months after admission, the patient was asymptomatic, with complete resolution of previous analytical and imagiologic findings, and antituberculous therapy was discontinued. No treatment side effects were registered during this period.

## Discussion

Disseminated TB is a severe rare form of TB, resulting from hematogenous or lymphatic dissemination of *M. tuberculosis* infection. Historically, the term miliary TB was coined in 1700 to describe diffusely infected lungs resembling millet seeds. Originally, it was used as a pathological term and, afterwards, as a radiographic description. It denotes all forms of progressive disseminated hematogenous TB that present with classic micronodular lung appearance, characteristic of hematogenous spread. The real incidence of miliary TB is unknown as its notification by the World Health Organization (WHO) and the United States Centers for Disease Control and Prevention (CDC) aggregates cases with both pulmonary and extrapulmonary involvement under pulmonary TB. However, the estimated incidence is reported as low as 2% of TB cases [5].

Miliary TB is more frequently seen in immunosuppressive states, as the risk for extrapulmonary TB increases as immunosuppression advances. However, patients without high-risk conditions may also present disseminated disease [6,7]. In addition to acquired or iatrogenic immunosuppression, medical conditions, such as pulmonary silicosis, have been associated with a higher risk for TB infection. Chronic silica exposure contributes to fibrotic lung tissue, decreased dendritic cell activation, and altered alveolar macrophage response, which play a key role, both in TB bacilli phagocytosis and inflammatory response [8-10]. These factors lead to increased susceptibility and severity of bacterial infections such as TB, especially with prolonged exposure [11].

Clinical manifestations of disseminated TB tend to be subacute or chronic, although fulminant presentations with multiorgan failure or septic shock can occur [4]. Diagnosis can be challenging as clinical presentation is frequently non-specific. Multiorgan involvement may impair diagnostic workup, especially if TB is not initially suspected. Appropriate diagnosis may be delayed or even missed in patients who do not present typical symptoms of pulmonary TB [12]. Furthermore, diagnostic analytic and bacteriological findings, in extrapulmonary sites, are difficult to obtain, thus hampering disseminated TB diagnosis.

Considering the aforementioned clinical case, PCR detection of *M. tuberculosis* was obtained in both sputum and urine samples, and there was histologic confirmation of renal involvement with glomerulonephritis. However, further documentation of both articular and pericardial involvement was not possible. Nevertheless, when considering this whole clinical presentation, it is almost impossible to dissociate articular, ganglionic, pericardial, and pleural involvement from disseminated TB diagnosis.

Supporting this assumption, we should take into account the low mycobacteriology yield throughout different fluids. For example, in pericardial fluid, acid-fast bacilli in culture can be identified in 56% of cases [13], while in pleural fluid and urine, the percentage of positive results can be as low as 20% [14,15]. When considering TB-associated glomerulonephritis, positive results are even rarer [15]. Nevertheless, the gold-standard method for TB diagnosis remains the cultural identification of *M. tuberculosis*, so fluid/tissue specimen collection is essential to increase diagnostic yield.

The usual treatment of miliary TB generally consists of the standard quadruple antimicrobial regimen, for a minimal time period of six months. Longer treatment, as in our patient, is conditioned by organ involvement, as the central nervous system and osteoarticular, renal, and ganglionic presentations usually require longer duration, with clinically individualized decisions [16].

## Conclusions

Disseminated or military TB is a rare condition that poses a hard diagnostic challenge for every clinician. In the absence of pulmonary symptoms, its non-specific presentation could delay or compromise diagnosis. Given its high incidence worldwide, disseminated TB should be included in the differential diagnosis of patients who present subacute/chronic multiorgan signs and symptoms, regardless of respiratory involvement. Special attention to immunosuppressive states is mandatory, along with known chronic medical illnesses that increase the risk for disseminated TB infection.

Extrapulmonary TB diagnosis, with pathogen identification in Lowenstein-Jensen cultures or *M. tuberculosis *DNA detection by PCR, is often difficult. Nevertheless, diagnosis should be swift, in order to early initiate antituberculous therapy. Additionally, this case points out that clinicians should be aware of heterogeneous disease progression, as initial detection of an organ involvement does not exclude possible further disseminated disease.

## References

[1] World Health Organization (2025). Global tuberculosis report 2024. Case notifications. https://www.who.int/teams/global-tuberculosis-programme/tb-reports/global-tuberculosis-report-2024/tb-diagnosis-and-treatment/2-1-case-notifications.

[2] World Health Organisation (2025). Global tuberculosis report 2023. TB incidence. 2023.

[3] Behr MA, Kaufmann E, Duffin J, Edelstein PH, Ramakrishnan L (2021). Latent tuberculosis: two centuries of confusion. Am J Respir Crit Care Med.

[4] Sydow M, Schauer A, Crozier TA, Burchardi H (1992). Multiple organ failure in generalized disseminated tuberculosis. Respir Med.

[5] Sharma SK, Mohan A, Sharma A, Mitra DK (2005). Miliary tuberculosis: new insights into an old disease. Lancet Infect Dis.

[6] Jones BE, Young SM, Antoniskis D, Davidson PT, Kramer F, Barnes PF (1993). Relationship of the manifestations of tuberculosis to CD4 cell counts in patients with human immunodeficiency virus infection. Am Rev Respir Dis.

[7] Golden M, Vikram HR (2005). Extrapulmonary tuberculosis: an overview. Am Fam Physician.

[8] Konečný P, Ehrlich R, Gulumian M, Jacobs M (2019). Immunity to the dual threat of silica exposure and mycobacterium tuberculosis. Front Immunol.

[9] Girdler-Brown BV, White NW, Ehrlich RI, Churchyard GJ (2008). The burden of silicosis, pulmonary tuberculosis and COPD among former Basotho goldminers. Am J Ind Med.

[10] Jamshidi P, Danaei B, Arbabi M (2025). Silicosis and tuberculosis: a systematic review and meta-analysis. Pulmonology.

[11] Lanzafame M, Vento S (2021). Mini-review: silico-tuberculosis. J Clin Tuberc Other Mycobact Dis.

[12] Heffner JE, Strange C, Sahn SA (1988). The impact of respiratory failure on the diagnosis of tuberculosis. Arch Intern Med.

[13] Reuter H, Burgess L, van Vuuren W, Doubell A (2006). Diagnosing tuberculous pericarditis. QJM.

[14] Gopi A, Madhavan SM, Sharma SK, Sahn SA (2007). Diagnosis and treatment of tuberculous pleural effusion in 2006. Chest.

[15] Sun L, Yuan Q, Feng J, Yao L, Fan Q, Ma J, Wang L (2012). Be alert to tuberculosis-mediated glomerulonephritis: a retrospective study. Eur J Clin Microbiol Infect Dis.

[16] National Collaborating Centre for Chronic Conditions (UK), Centre for Clinical Practice at NICE (UK) (2011). Tuberculosis: clinical diagnosis and management of tuberculosis, and measures for its prevention and control. Tuberculosis: Clinical Diagnosis and Management of Tuberculosis, and Measures for Its Prevention and Control.

